# Assessment of Unconscious Decision Aids Applied to Complex Patient-Centered Medical Decisions

**DOI:** 10.2196/jmir.3739

**Published:** 2015-02-05

**Authors:** Andrew Wilhelm Manigault, Ian Michael Handley, Summer Rain Whillock

**Affiliations:** ^1^Montana State UniversityDepartment of PsychologyBozeman, MTUnited States; ^2^Ohio UniversityPsychology DepartmentAthens, OHUnited States

**Keywords:** unconscious, thought, intuition, medical, decision, judgment, cognitive load, patient-centered

## Abstract

**Background:**

To improve patient health, recent research urges for medical decision aids that are designed to enhance the effectiveness of specific medically related decisions. Many such decisions involve complex information, and decision aids that independently use deliberative (analytical and slower) or intuitive (more affective and automatic) cognitive processes for such decisions result in suboptimal decisions. Unconscious thought can arguably use both intuitive and deliberative (slow and analytic) processes, and this combination may further benefit complex patient (or practitioner) decisions as medical decision aids. Indeed, mounting research demonstrates that individuals render better decisions generally if they are distracted from thinking consciously about complex information after it is presented (but can think unconsciously), relative to thinking about that information consciously or not at all.

**Objective:**

The current research tested whether the benefits of unconscious thought processes can be replicated using an Internet platform for a patient medical decision involving complex information. This research also explored the possibility that judgments reported after a period of unconscious thought are actually the result of a short period of conscious deliberation occurring during the decision report phase.

**Methods:**

A total of 173 participants in a Web-based experiment received information about four medical treatments, the best (worst) associated with mostly positive (negative) side-effects/attributes and the others with equal positive-negative ratios. Next, participants were either distracted for 3 minutes (unconscious thought), instructed to think about the information for 3 minutes (conscious thought), or moved directly to the decision task (immediate decision). Finally, participants reported their choice of, and attitudes toward, the treatments while experiencing high, low, or no cognitive load, which varied their ability to think consciously while reporting judgments. Cognitive load was manipulated by having participants memorize semi-random (high), line structured (low), or no dot patterns and recall these intermittently with their decision reports. Overall then, participants were randomly assigned to the conditions of a 3 (thought condition) by 3 (cognitive-load level) between-subjects design.

**Results:**

A logistic regression analysis indicated that the odds of participants choosing the best treatment were 2.25 times higher in the unconscious-thought condition compared to the immediate-decision condition (*b*=.81, Wald=4.32, *P*=.04, 95% CI 1.048-4.836), and 2.39 times greater compared to the conscious-thought condition (*b*=.87, Wald=4.87, *P*=.027, 95% CI 1.103-5.186). No difference was observed between the conscious-thought condition compared to the immediate-decision condition, and cognitive load manipulations did not affect choices or alter the above finding.

**Conclusions:**

This research demonstrates a plausible benefit of unconscious thinking as a decision aid for complex medical decisions, and represents the first use of unconscious thought processes as a patient-centered medical decision aid. Further, the quality of decisions reached unconsciously does not appear to be affected by the amount of cognitive load participants experienced.

## Introduction

### Background

To improve or maintain patient health and well-being, it is of course important that patients and/or health-care providers make the best (or at least beneficial) decisions regarding treatment options, behaviors, diagnoses, test options, and so forth. Complicating this, however, these individuals must commonly consider very complex information to make medical decisions. Thus, growing research has investigated decision aids that might benefit patients and health-care providers as they consider complex medical information [1]. Decision aids are generally used to engage both patients and practitioners in the decision process, and allow patients to understand the potential risks and benefits of a given medical choice. Additionally, decision aids often incorporate value clarification exercises to help patients consider the personal values they place on potential risks and benefits. For example, patients may go through a list of values, select the five most important ones, and bear those in mind while making a medical decision. De Vries et al [1] suggest that decision aids can make use of deliberative processes (requiring intentional and analytical thinking) or intuitive processes (which are more affective, unconscious, or automatic) to consider decision-relevant information. Although decision aids that make use of deliberative processes are more common, both types of processes have strengths and weaknesses for medical decisions. Regarding this, De Vries et al warn that decision aid developers “should be aware that the current common practice to encourage patients to extensively analyze available choice options, typically immediately after information exposure, lacks solid theoretical and empirical grounding…and may even have some harmful side effects to preference construction processes” (p. 159 [1]) and suggest that optimal decision aids would take advantage of the complementarity of the two systems [1]. Consistent with this later suggestion, the current paper explores the possibility that unconscious-thinking processes [2]—which theoretically incorporate intuitive processes as well as more time-demanding and analytic unconscious deliberation processes [3-5]—can provide patients with a valuable decision aid for complex medical information.

According to Dijksterhuis and Nordgren [2], unconscious thought is the “object-relevant or task-relevant cognitive or affective thought processes that occur while conscious attention is directed elsewhere” (p. 96 [2]), whereas conscious thought involves these same processes, but within conscious awareness. They suggest that unconscious thinking processes make use of vast mental resources, whereas conscious thinking processes rely on limited resources such as working and short-term memory. Thus, they argue, individuals often arrive at better decisions from complex information when they process information unconsciously (while conscious thinking processes are distracted from the relevant decision task). Much research supports this possibility [2,6-11], and generally follows a paradigm established by Dijksterhuis [6]. In this paradigm, individuals receive much information about 3-4 targets (eg, roommates). Further, one target is associated with mostly positive attributes, one with mostly negative attributes, and the other(s) with a balance of positive and negative attributes. Next, participants report their decisions regarding, or preferences for, targets either immediately (allowing for minimal conscious or unconscious thinking), after 3 minutes in which they think about the presented information (ie, think consciously), or after 3 minutes in which they engage in an unrelated task that distracts them from thinking consciously about the presented information (but can still think unconsciously). Typical results demonstrate an “Unconscious Thought Effect” (UTE) such that participants in the “unconscious thought” condition arrive at better decisions (prefer the best over the worst target) relative to participants in the immediate-decision condition. As well, participants in conscious-thought conditions often arrive at decisions comparable to those in the immediate-decision conditions.

Since Dijkterhuis’s first article reporting the UTE [6], much research has either replicated the effect or called the effect into question. A recent Bayesian meta-analysis of 16 studies conducted by Newell and Rakow [12] does not support the existence of the UTE, and a study by Huizenga and colleagues [13] provides additional evidence against the merits of unconscious thought processes. Conversely, a meta-analysis of 92 studies conducted by Stick et al [14] suggest that the UTE is modest but reliable, and research by Creswell and colleagues [15] provides strong fMRI (functional magnetic resonance imaging) evidence consistent with the UTE. Creswell and colleagues addressed important critiques of the UTE by providing physiological evidence that the UTE relies on specific neural reactivation to occur, and that conscious and unconscious thought processes recruit non-overlapping neural regions [15]. Nevertheless, when considering unconscious thought as a potential decision aid, developers ought to consider the present paper as part of a growing literature that deserves a thorough review before justifying any reforms.

The meta-analysis conducted by Strick et al [14] demonstrated that the UTE is stronger when the presented information is complex, the goal to make a decision is emphasized and formulated in a holistic fashion, and the decision task is ecologically valid. Thus, unconscious thought may be particularly suited to aid sound medical decisions; medical decisions are typically complex (eg, involving a large number of trade-offs between length and quality of life [1]), patients and health-care providers are generally motivated to find the best course of treatment, and the outcomes of such decisions bear real-life consequences that ensure a level of ecological validity. In fact, an experiment by DeVries and colleagues investigated the UTE in a health context, and demonstrated that in-training clinical-psychologists (graduate students) achieve more accurate psychiatric diagnoses following a period of unconscious versus conscious thinking. However, the present research sought to investigate the potential benefit of unconscious thinking as a decision aid for the broad population of patients (and is the first to our knowledge to do so), without specific health or medical training. This is noteworthy because experts within a given decision domain (eg, training clinicians) demonstrate the UTE more than non-experts [16] (eg, patients and the lay-public generally). Thus, demonstrating this effect on medically related decisions—relative to a “non-thinking” control group—even among a general sample could reveal that unconscious thought is a useful decision aid for making complex medical decisions that affect patient health and well-being. Further, the reported research addresses a potential methodological criticism of past unconscious-thought research: participants could theoretically think consciously while reporting their decisions (ie, during the decision phase of the experiment), even following a period of distraction. If true, the UTE might actually result from conscious thinking processes. We explore this possibility by manipulating the amount of cognitive load participants experience during the decision phase of the experiment. This is a relevant and ecologically valid manipulation given the cognitively demanding context of many medical environments, and is novel within the unconscious-thought literature.

### Current Aims, Experiment Overview, and Hypotheses

The aim of the current research was to test whether patient-centered decisions regarding complex treatment options are better following a period of unconscious thought relative to immediate decisions, indicating unconscious thought can be a beneficial decision aid. For this initial investigation, a Web-based sample of participants received a cover story entailing “their” recent hospital admission and diagnosis. Next, all participants received side-effect/attribute information for four potential treatments, one of which was the best, one the worst, and two of which were in the middle. Following this, participants were randomly assigned to a thought condition in which they completed a distraction task for 3 minutes (unconscious), deliberated for 3 minutes (conscious), or were given no time (immediate decision), before reporting their judgments about the treatments. Finally, participants rendered their judgments while under a high, low, or no cognitive load. Thus, overall, participants were randomly assigned to the conditions of a 3 (thought condition) by 3 (cognitive-load level) between-subjects design. Participant’s choice of treatment, and attitude ratings of each treatment, were recorded.

The primary hypothesis was that we would observe a UTE such that participants in the unconscious-thought condition (but not participants in the conscious-thought condition) would choose the best treatment relative to the immediate-decision condition (control group). This same effect was predicted for participants’ treatment attitudes, although this measure is less critical than investigating actual treatment choices; the choices patients and health practitioners make tend to be more consequential to health outcomes than their attitudes toward various treatments. Additionally, we predicted that participants in the unconscious-thought condition would choose (and form more favorable attitudes toward) the best treatment relative to the conscious-thought condition. Although this is not critical to demonstrating the UTE per se, this prediction is consistent with much of the unconscious-thought literature [14], and speaks directly to the possibility that unconscious thought may be an effective decision aid relative to purely deliberative decision aids.

Further, we propose two competing exploratory hypotheses regarding the effect of cognitive load during the decision phase. First, if participants in the unconscious-thought condition actually generate their decisions consciously during the decision phase, then their decisions should become worse as cognitive load increases, and the UTE should only manifest under no- and low-load conditions. Second, if participants in the unconscious-thought condition truly generate their decisions unconsciously, then their decisions should be comparable across load conditions, and the UTE should occur unaffected by cognitive load.

## Methods

### Participants

A total of 173 Amazon Mechanical Turk workers participated in this Web-based experiment. The Institutional Review Board of the Montana State University approved all procedures in advance. Participants were compensated with US $0.50, and (retroactively) an additional $1.00 bonus for choosing the best treatment option. Participants were 87 males (50.3%) and 86 females (49.7%) with ages ranging from 18 to 73 years (mean 28.38, SD 8.18). One participant reported that he/she was 2 years old (we assumed this was a “typo” and the individual intended to report an age in the twenties); excluding this participant from the analyses had no effect on the results. In total, 135 participants categorized their ethnicity as White/Caucasian (78.0%), 12 as Black/African American (6.9%), 11 as Asian (6.4%), 3 as Native American (1.7%), and 12 as “other” (6.9%). Further, we analyzed these participant characteristics independently as a function of the independent variables, and found no significant effects (all *P*s>.15). Thus, our random assignment procedure succeeded at distributing participants evenly across the experiment conditions.

### Materials

Treatment side-effects/attributes were first pretested using a separate sample of 52 Amazon Mechanical Turk workers. Participants from this sample rated each of 75 side-effects/attributes on valence and importance. Specifically, participants were asked to “Rate the following side effect in terms of how positive/negative it is”, then were randomly shown one of the 75 side-effects/attributes and responded on a 9-point scale ranging from 1 (very negative) to 9 (very positive). Following, participants were shown the same side-effect/attribute and asked to “Rate the following side effect in terms of how important it is” on a 9-point scale ranging from 1 (very unimportant) to 9 (very important). Of note, positive side effects/attributes in the present design were independent of the intended treatment effects (to cure the patient), but were considered to be positive or beneficial.

A total of 35 (17 positive and 18 negative) side-effects/attributes were selected as stimuli for the current experiment (see Multimedia Appendix 1 for selected side-effects/attributes and pre-test ratings). We chose side-effects/attributes with moderate pre-test ratings on both valence and importance dimensions to ensure that one or a few side-effects/attributes would not dominate choices, thereby oversimplifying the decision process. Using these pre-test data, we then assessed the actual quality of each treatment that was used in the main experiment by weighting the valance of treatment side-effects/attributes by importance. This follows logically from research conducted by Bos and colleagues [5], which demonstrates that unconscious thought makes use of valance and importance information in a logical way (approximately weighting valance by importance) to make sound decisions. For example, a highly positive side-effect/attribute of low importance can affect decisions less than a mildly positive side-effect/attribute of high importance. Thus, for each treatment, we first multiplied the valance rating by the importance rating for each positive side-effect/attribute, then summed these products. We did this again for each negative side-effect/attribute, independently for each treatment. Next, for each treatment, we subtracted the resulting sum for the negative side-effects/attributes from the sum for the positive side-effects/attributes. This created a “quality rating” for each treatment (more positive numbers indicate higher quality), which was analyzed using contrasts within a repeated-measure ANOVA (analysis of variance). These contrasts confirmed that the best treatment was viewed as having better quality (mean 297.75, SD 139.32) than the two balanced treatments (mean 128.92, SD 110.67 and mean 137.48, SD 117.76; *F*_1,51_=366.95, *P*<.001), and the worst treatment was viewed as having lower quality (mean=−48.71, SD 88.73) than the two balanced treatments (*F*_1,51_=308.51, *P*<.001). Further, the two balanced treatments were rated comparably, *F*<1.

### Procedure

#### Internet Sample and Platform

Participants were recruited through Amazon Mechanical Turk (a Web-based crowdsourcing marketplace), and redirected to Qualtrics (a survey website) to complete the experiment. Amazon Mechanical Turk users were eligible to participate if they resided in the United States, had completed over 100 remunerated tasks (known as Amazon Mechanical Turk HITS), and had an approval rating over 90% (meaning that 90% of tasks completed by users where deemed worthy of remuneration by previous employers). Data collection started on April 30, 2014 and finished on May 9, 2014; participants had the option of leaving the experiment at any time but were unable to return to previous pages. A completeness check was automatically recorded by Qualtrics (dependent upon viewing the last page of the survey), and the completion rate was 59.6% (173/290). Incomplete surveys were not included in the final analyses. Amazon Mechanical Turk’s account registration system was used to prevent multiple entries (a given account could only complete the experiment once). The average time of survey completion was 13.5 minutes; no atypical timestamp surveys were excluded.

#### Experiment Flow

Participants were first presented with a consent form, completed a demographic questionnaire, and then read the following scenario:

Please imagine yourself as a recently admitted patient at a hospital. The doctors have diagnosed you with a Campylobacter infection. They then present you with different treatment options which all have a large number of positive and negative side effects. Since all of the treatments will treat the Campylobacter infection, the only basis for comparison are their associated side effects. Also, given the progression of the infection, a decision must be taken in the next few minutes. This part of the experiment is concerned with the way in which we form an impression on the basis of a number of attributes. In a few moments you will be presented with four treatments along with side effects that each of the treatments possess. Please read these sentences carefully, study each one until the next appears. Later, we will ask you a series of questions concerning the impressions that you have formed of the four different treatments.

Following, participants were told that choosing the best treatment option would grant them a $1 bonus. The possibility for bonus remuneration helped ensure that participants were motivated to choose the best treatment. Subsequently, each participant was sequentially shown 48 side-effects/attributes in random order. Each side-effect/attribute was presented for 4 seconds and attributed to one of four treatment options. Overall, one option was best (8 positive and 4 negative side-effects/attributes), one was worst (4 positive and 8 negative side-effects/attributes), and the other two were balanced (6 positive and 6 negative side-effects/attributes). This type of stimuli presentation was used to ensure decision complexity following past research methods [6-8]. Next, participants were randomly assigned to either an unconscious-thought, conscious-thought, or immediate-decision condition. Finally, participants were randomly assigned to render their treatment choices and attitudes while experiencing high, low, or no cognitive load (details for all manipulations provided below). Last, participants were debriefed, thanked for their participation, and given a code to redeem compensation through Amazon Mechanical Turk.

### Independent Variables

#### Thought Condition

After participants received all of the side-effect/attribute information, they were randomly assigned to one of three thought conditions. Participants in the unconscious-thought (or distraction) condition were instructed to complete as many anagrams as they could within 3 minutes, and were presented with a list of 36 anagrams. This task is commonly used in unconscious-thought experiments to consume and distract conscious thought, yet allows unconscious thought to continue processing decision-relevant information. Participants in the conscious-thought condition read the following instructions: “For the next 3 minutes, consider the four different treatments and the side effects you read about. Think about which treatment is the best and/or which treatment you like the most. Try to only think of the treatments and which treatment you might personally prefer.” Thus, these participants were specifically instructed to think consciously about the side-effect/attribute information, and had time to do so. Finally, participants in the immediate-decision condition directly moved on to the judgment task, and had insufficient time to think consciously or unconsciously about the side-effect/attribute information.

#### Cognitive Load

**Figure 1:**
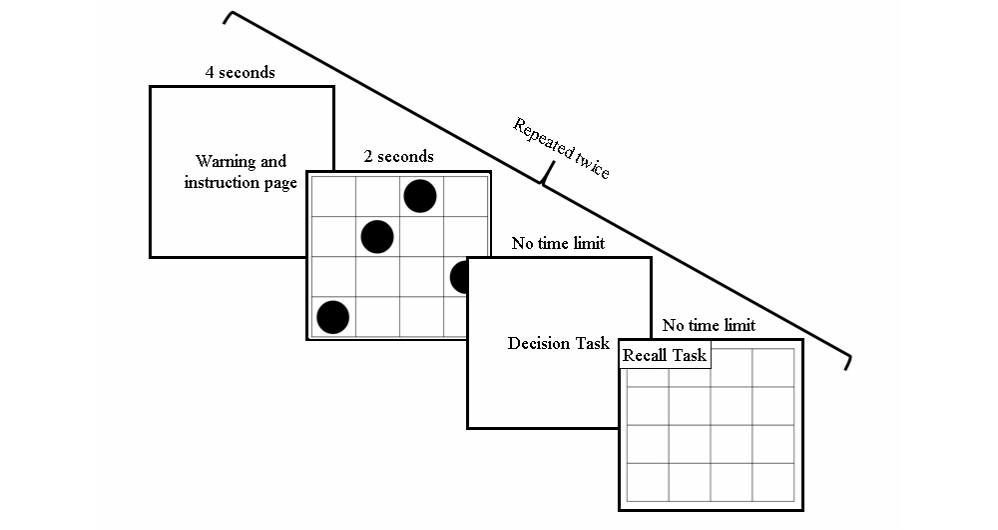
Flow of the decision phase. The first decision task consisted of the choice variable while the second decision task consisted of the attitude rating. The high cognitive load is displayed for illustrative purposes; the actual pattern depended on cognitive load condition.

Cognitive load was manipulated using 4 x 4 matrices with 4 dots presented within 16 possible locations. The manipulation stimuli were modeled after Haymen et al [17], who demonstrated their effectiveness in producing high or low cognitive load. High cognitive-load manipulations consisted of a semi-random scatter, whereas the low cognitive-load manipulation consisted of a 4 dot line (see Multimedia Appendix 2 for actual stimuli). Participants were instructed to memorize the exact pattern and warned that they would later be asked to reproduce it. Ultimately, participants reported judgments about the treatments interspersed with the load manipulations. Specifically, the decision phase entailed a repeated sequence of events: pattern exposure, treatment choice, pattern recall, new pattern exposure, treatment evaluations, pattern recall (see Figure 1). Participants in the no cognitive-load condition were only presented with the judgment tasks.

We reasoned that the semi-random dot patterns were difficult to memorize and that participants in the high cognitive-load condition would not be able to consciously process the side-effects/attributes information and rehearse the dot pattern as they were reporting their choice/attitude ratings. Conversely, the linear dot patterns (low cognitive load) would require little active cognition to maintain in working memory and therefore could allow participants to engage in conscious deliberation.

### Dependent Measures

#### Choice

Participants were asked to make two main treatment judgments. First, participants were instructed to: “Choose a treatment” and could click on one of the four treatment options. This is the primary dependent measure, as this choice directly affects patient health outcomes and experienced side-effects. For this measure, participants who chose the best treatment option were scored “1”, whereas participants who chose any of the other options were scored “0”.

#### Attitude Measure

Next, participants separately rated each of the four treatments (eg, “Your impression of Treatment A was…”) on scales ranging from −25 (very negative) to 25 (very positive). Following previous research in the unconscious-thought literature, participants’ rating for the worst treatment was subtracted from their rating for the best treatment, resulting in an attitude preference measure. Higher numbers on this measure indicate a more positive rating for the best over the worst option.

## Results

### Manipulation Check

On average, participants in the low cognitive-load condition correctly recalled significantly more dots (mean 6.91, SE 0.29) than participants in the high cognitive load condition (mean 5.73, SE 0.25; *t*_110_=−3.031, *P*=.003). This worse recall in the high versus low cognitive-load conditions supports the idea that the pattern task was more cognitively demanding than in the former, and is consistent with the results and interpretation of Heyman et al [17].

### Dependent Variables

#### Choice

We conducted a hierarchical logistic regression to predict participants’ choice for the best treatment, entering Thought Condition as a predictor in the first step (given this was a theoretically important variable), Cognitive-Load Condition as an additional predictor in the second step (given it was exploratory), and the interaction between these two factors as yet an additional predictor in the third step. As hypothesized, the first step revealed a main effect of thought condition; this predictor demonstrated a significant improvement over the constant-only model (*χ* ^*2*^_2_=6.39, *P*=.04, Nagelkerke *R* ^*2*^=.05), and this single-predictor model fit the data well (for the Hosmer and Lemeshow test, *P*=.81). Further, there was no main effect of cognitive-load condition, nor a Thought Condition by Cognitive-Load Condition interaction. Adding Cognitive-Load in the second step revealed no improvement in the model (*χ* ^*2*^_2_=1.54, *P*=.46), nor did adding the interaction term in the third step (*χ* ^*2*^_4_=3.83, *P*=.43). Given this, we interpreted the main effect of thought condition based on the first step of the analysis. Demonstrating a UTE, the odds of participants choosing the best treatment were 2.25 times higher in the unconscious-thought condition compared to the immediate-decision condition (*b*=.81, Wald=4.32, *P*=.04, 95% CI 1.048-4.836), and 2.39 times greater in the unconscious-thought condition compared to the conscious-thought condition (*b*=.87, Wald=4.87, *P*=.027, 95% CI 1.103-5.186). Further, the odds of choosing the best treatment were comparable across the conscious-thought and immediate-decision conditions (*b*=−.06, Wald=0.22, *P*=.88; for the constant, *b*=−.88, Wald=9.31, *P*=.002). Overall, thought condition was the only significant predictor of choice, and participants in the unconscious-thought condition demonstrated a higher probability of making the correct choice versus the control condition (see Table 1 for choice contingency table). Further, participants overall found the decision task quite complex, given 112 of 173 participants (64.7%) chose an incorrect treatment option. This is important given UTEs tend to manifest in complex decision tasks.

We also explored choice as a function of cognitive-load condition separately for each thought condition. None of these analyses demonstrated a significant effect of cognitive load (all *X* ^*2*^< 2.56, all *Ps*>.05).Of note, if correct decisions arising from unconscious thought actually result from conscious thought during the decision phase, then higher levels of load should result in worsening decisions within the unconscious thought conditions. But, if anything, load resulted in better choices in the unconscious thought conditions (see Table 1). Nonetheless, so as to fully assess the interaction of cognitive load and thought conditions, we also reanalyzed our data after combining the immediate and conscious-thought conditions (unconscious thought vs others), and after combining the low- and high-load conditions (no load vs load). Again, this analysis revealed an effect of thought condition (*b*=.84, Wald=6.34, *P*=.012, 95% CI 1.205-4.463), and again the effect of load condition and the interaction of thought and load were not significant (all *Ps*>.28). Further, we found the power of our experiment to be .73 for this simplified interaction. Thus, our experiment had reasonable power to detect an effect of load on the UTE.

**Table 1:** Contingency table for choice broken down by thought condition and cognitive-load condition (n=173).

Thought Condition	Cognitive Load	Choice		Totals
		Correct	Incorrect	
**Unconscious**				
	No Load	10	13	23
	Low Load	10	8	18
	High Load	8	9	17
	Total	28 (48.3%)	30 (51.7%)	58
**Immediate**				
	No Load	3	9	12
	Low Load	4	20	24
	High Load	10	12	22
	Total	17 (29.3%)	41 (70.7%)	58
**Conscious**				
	No Load	7	19	26
	Low Load	4	10	14
	High Load	5	12	17
	Total	16 (28.1%)	41 (71.9%)	57

#### Attitude Measure

The attitude measure was analyzed using a 3 (thought condition) by 3 (cognitive load condition) between-subjects ANOVA. This analysis revealed no significant effects (all *F*s<1.42), and no planned comparisons were significant (*t*s<1).

## Discussion

### Principal Findings

In this experiment, participants who were distracted for 3 minutes after receiving treatment information—and thus had the opportunity to think unconsciously—were significantly more likely to choose the best treatment option relative to participants who made their choices immediately following the information (or thought consciously). To our knowledge, this is the first replication of the UTE using a patient-oriented medically related decision task. Further, no such advantage was observed for participants who consciously thought about the information for 3 minutes after receiving treatment information. These trends were mirrored in participants’ attitudes toward the best versus worst treatment, although not significantly. In the field, however, the choices patients and health practitioners make are more consequential to health outcomes than their attitudes toward various treatments. The choice results are strongly in line with a growing body of evidence demonstrating that individuals are more likely to make the best decisions when they think unconsciously, provided the decision task is complex, they are motivated to be accurate, and the task has ecological validity. These conditions were met in the current research: the task was complex and most participants chose an incorrect treatment, participants were motivated to choose the correct treatment (with a US $1 incentive), and the task was constructed to represent a real-life medical decision (albeit, with fictitious treatment information and a fictitious medical condition). Further, these conditions are clearly met in many medical contexts, as treatment information is often complex and all parties involved are motivated to arrive at correct treatment choice. Thus, unconscious thinking processes may greatly aid decision making within many medical contexts.

The present research also explored the possibility that UTEs are not actually the result of unconscious thinking that occurs while people are distracted, but of conscious processes that occur while people solidify and report a judgment. And, to our knowledge, this is the first experiment to investigate this potential alternative account for the UTE. To test this possibility, we varied the cognitive load participants experienced while they reported their choice and attitude judgments, and this manipulation was successful. If the UTE actually results from conscious thinking at the time of judgment, then participants in the unconscious-thought conditions should do worse if they experience high load (compared to low or no load) while reporting their judgments. However, the cognitive-load manipulation had no effect on either of the dependent measures, nor did it interact with thought condition. This overall null effect of cognitive load suggests the UTE does not result from conscious processing at the time of judgment, and judgments are accessed with negligible effort during the decision phase.

### Limitations and Future Directions

The reported results indicate that unconscious thought may serve as a beneficial decision aid for patients facing complex medical decisions. But, of course, this initial investigation has limitations and we encourage further research into unconscious thinking in medical contexts before advocating any decision-making reform. Foremost, the tested decision task involved an imaginary scenario and not a personal health event. Still, participants were motivated to arrive at the correct decision and the greater motivation real patients likely experience should theoretically enhance the UTE [5]. Related, the stimuli used in this experiment were fictitious, although designed to appear medically relevant. Future research should employ real information as the basis of decisions and focus on adapting the unconscious thought paradigm to real-life examples such as the trade-offs between length and quality of life faced by older or terminally ill populations or the time-sensitive medical decisions that one may face in an emergency room. Furthermore, the side-effect/attribute information was presented randomly rather than organized by treatment as would normally happen. This was important to create the decision difficulty needed to verify the results stemmed from unconscious, and not conscious, processes in this initial investigation. However, given the present results, future research could further test the benefit of unconscious thought as a decision aid under more realistic informational settings. Finally, we tested our predictions using a geographically dispersed Web-based sample, not with participants in a controlled environment, and cannot ensure strict instruction compliance. An exact laboratory replication could easily address this limitation. At the same time, however, the present results demonstrate the UTE for medical decisions using a Web-based sample, and suggest that a Web-based platform could be used to create decision aids that foster unconscious thinking.

Ultimately, it will be critical to demonstrate the UTE in actual medical contexts with participants facing real decisions for themselves or others. As of now, the present research reveals a plausible benefit of unconscious thought as an aid for patients’ medical decisions and future research will have to confirm that benefit in commonplace settings, with real information, and with vested decision-makers.

The present claim that unconscious judgments come to awareness relatively independently of cognitive load entails several limitations. First, the cognitive load manipulation used in the present experiment was comprised of visuospatial dot patterns whereas the decision task was primarily verbal. To the extent that visuospatial and verbal processing may employ different cognitive resources, it is possible that the present cognitive load manipulation did not interfere with the decision task enough to adequately test our hypotheses. Although visuospatial stimuli were preferred in the present study to help ensure instruction compliance (reporting random dot patterns requires memory processes because they are difficult to reproduce or write down), future studies should make use of verbal cognitive-load manipulations to address this issue. Additionally, participants were instructed to memorize dot patterns and report their judgments intermittently, but were not instructed to focus primarily on one task or the other. As such, participants had the opportunity to neglect the cognitive task so as to minimize its impact on their performance on the decision task. This eventuality may account for the difference in mean recall observed between the high and low cognitive-load conditions (of note, however, this difference replicates Heyman et al, who interpreted these differences as indicating greater task difficulty rather than decreased task compliance). Future studies could address this issue by specifically stating the primary and secondary task in the instructions. Finally, a baseline assessment of performance on the cognitive load task could also be implemented to assess the extent to which it is affected by the decision task. As ours is the first experiment to explore the influence of cognitive load during the decision phase on UTEs, we strongly encourage future research to address these issues to better couch our current findings.

### Conclusion and Implications

Patients and health practitioners alike commonly consider a vast amount of information to reach optimal medical decisions. Unfortunately, considerable evidence now indicates that conscious processes can be ill-equipped to integrate complex information, at least without aids (eg, notes, computers, etc). Quite simply, people cannot consciously retain and process vast amounts of information, and thus often form poor decisions via conscious processes. But, according to Dijksterhuis and Nordgren [2], unconscious thinking can process vast amounts of information with just a little time (eg, 3 minutes), and thus somewhat counterintuitively, individuals often come to better decisions when they are distracted from consciously considering decision-relevant information. The current research demonstrates this can also be true for patient-centered medical decisions.

A hypothetical (and relatively long-term) implication of the current research lies in the type of media used to test the UTE. Unconscious thought research readily utilizes computer and research software platforms, and a Web-based platform was used presently. Demonstrating the UTE in this fashion may constitute evidence that unconscious-thinking decision aids and value clarification exercises may be integrated with Web and mobile technologies within health care realms (given that current limitations are addressed of course). For example, a Web or mobile phone app may in some instances present the relevant information to a medical decision maker, provide a timed and cognitively consuming distraction, then solicit a decision. That is, it is conceivable that a Web or mobile app could model the stages used in the current research for real medical-decision tasks in a way that fosters unconscious thought, and thus better decisions. Further, the merits of unconscious thought should not be limited to treatment side-effects/attributes. Personal values could (theoretically) also be processed unconsciously. This implication is particularly relevant because unconscious thought can process decisional factors that are difficult to articulate or too numerous to maintain in conscious awareness [1-3]. Patients may therefore be able to use an “unconscious thought mobile app” as a beneficial value clarification exercise and successfully incorporate numerous personal values in their decision.

Finally, unconscious thinking may instill further benefits in medical contexts. First, other research demonstrates that individuals are more satisfied with the choices they make via unconscious thinking [7]. Thus, patients might experience more satisfaction with a treatment, and thus better adhere to it, if they chose that treatment following unconscious thought. Second, we investigated mock-patient decisions, but health practitioners might experience the greatest benefit from unconscious thought for complex medical decisions. For instance, research by Dijksterhuis and colleagues [8] showed that participants with more (vs little) expertise in a domain reach higher quality judgments after a period of unconscious thought. Thus, medical experts may realize the most advantage for choosing optimal treatments or generating accurate diagnoses in the face of complex and numerous symptoms, complications, side-effects, and risks, and some research already supports this possibility [16]. Given this, exploring the UTE in medical decision making for health care providers and patients has the potential to greatly and broadly enhance patient health and well-being.

## Multimedia Appendix 1

Treatment side effects and relative mean (M) and standard derivation (SD) ratings of valence and importance.

## Multimedia Appendix 2

High and low cognitive load dot patterns.

## Abbreviations

ANOVA: analysis of variance
UTE: unconscious thought effect
