# Supplementary figures and images for: Assessment of Unconscious Decision Aids Applied to Complex Patient-Centered Medical Decisions
Source: J Med Internet Res. 2015 Feb 5;17(2):e37. doi: 10.2196/jmir.3739 (PMC4342682; doi:10.2196/jmir.3739)

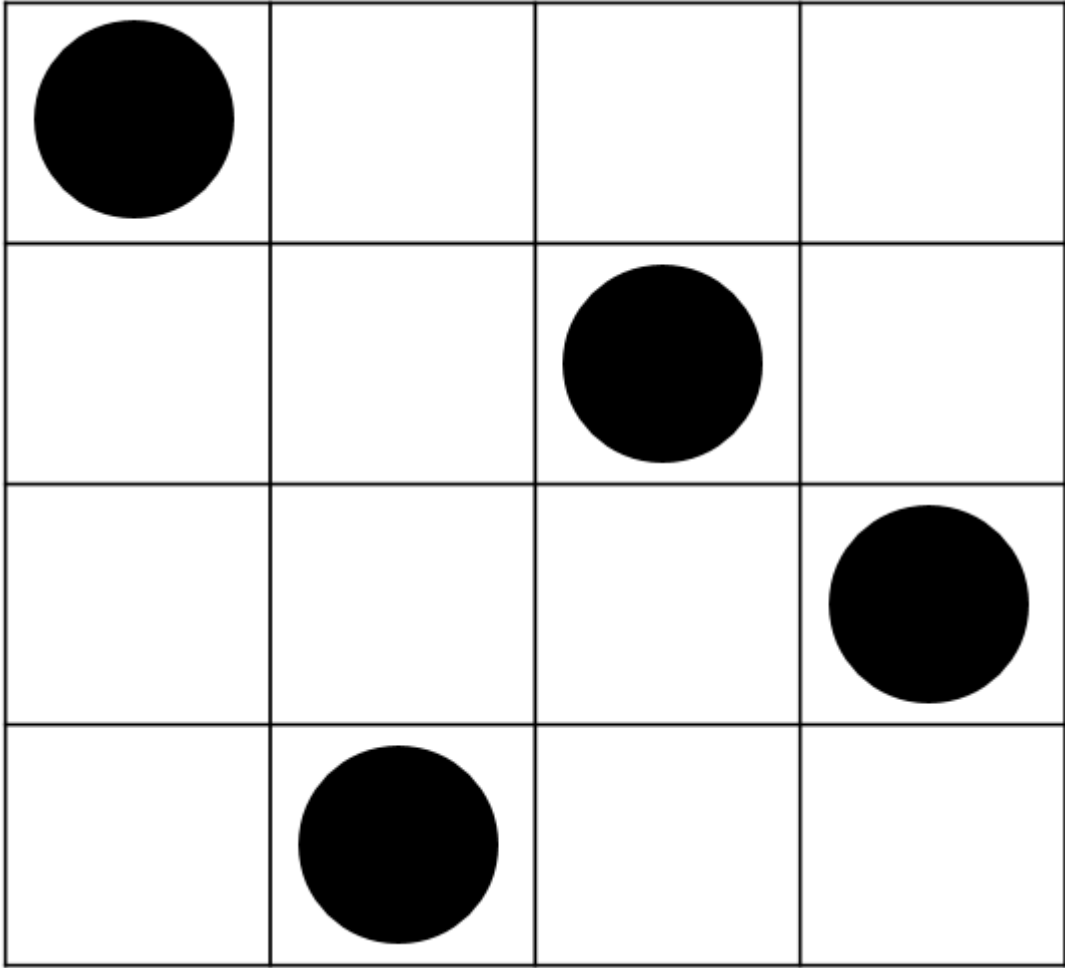

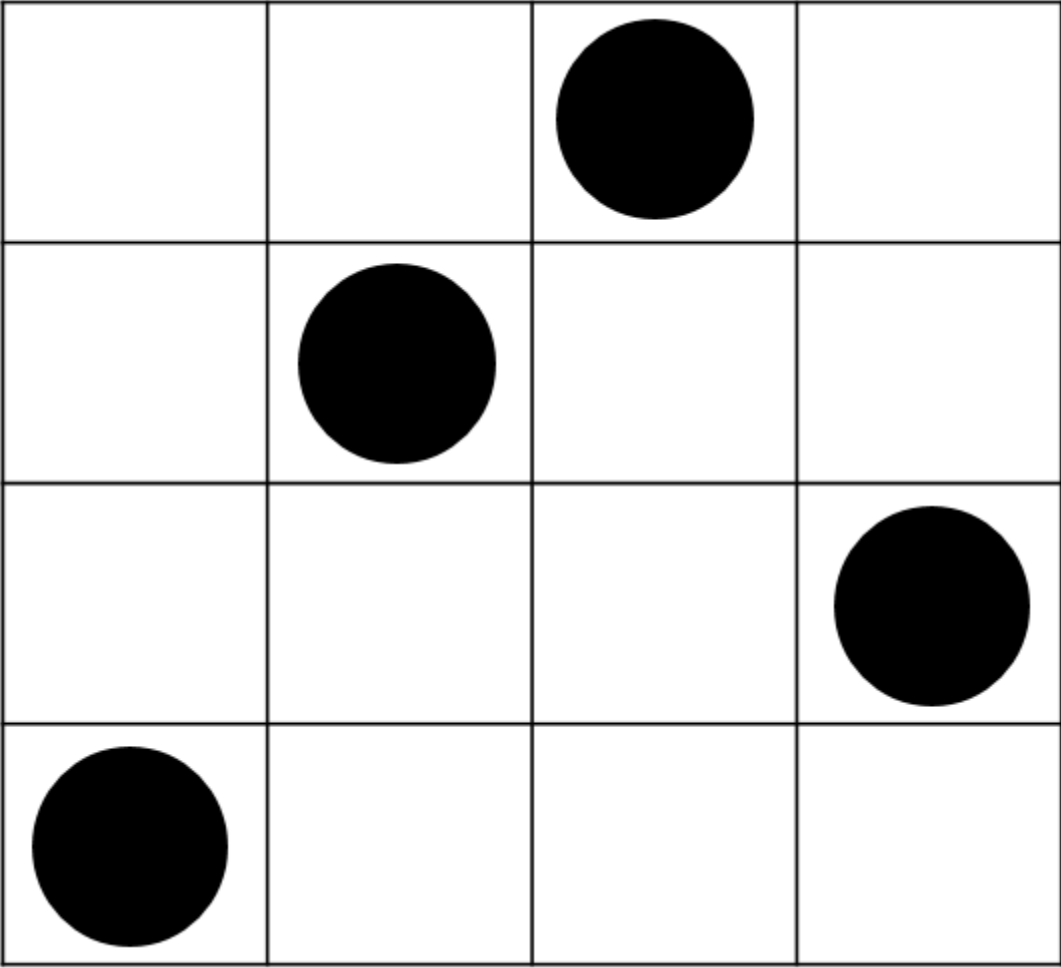

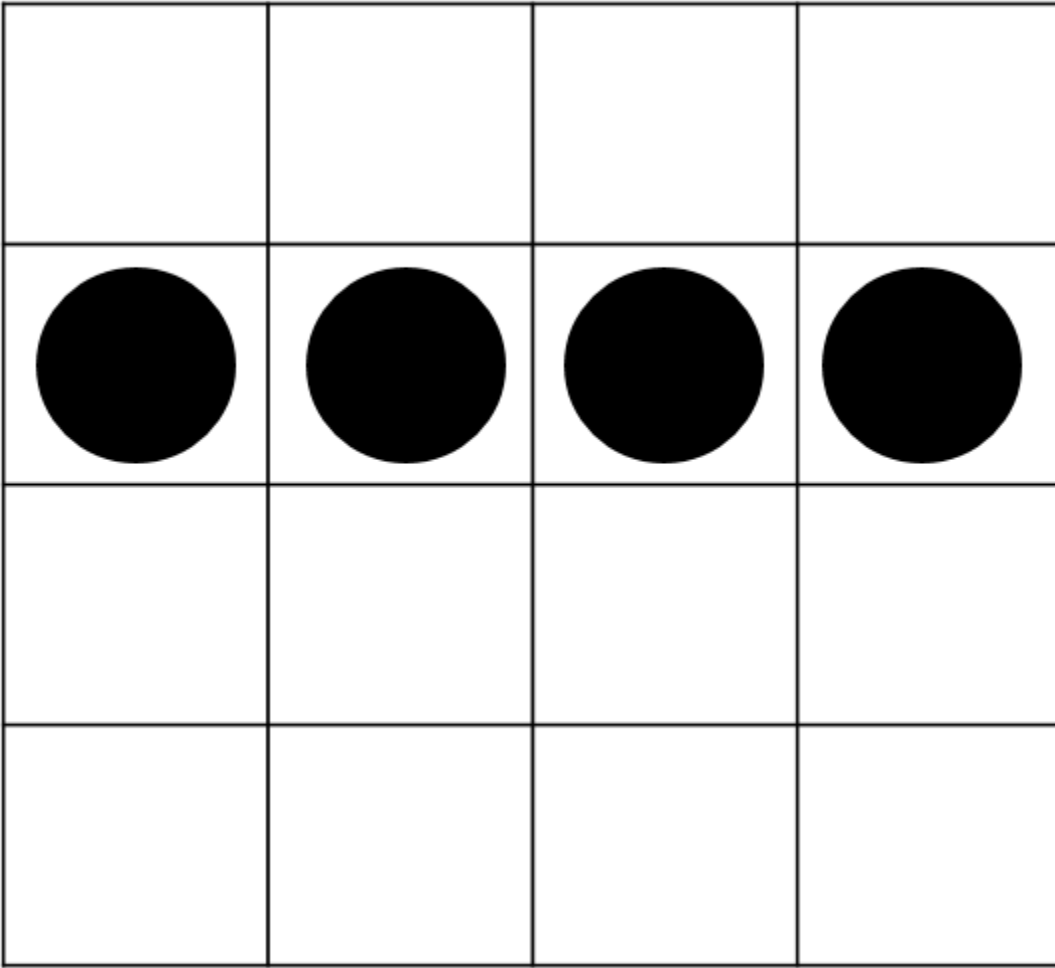

|  |                                                                                    |  |  |
|--|------------------------------------------------------------------------------------|--|--|
|  | 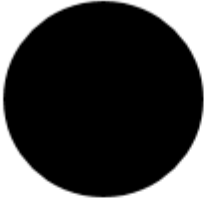  |  |  |
|  | 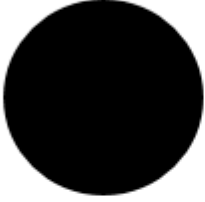  |  |  |
|  | 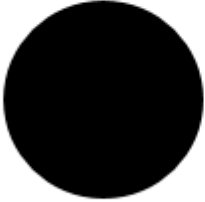  |  |  |
|  | 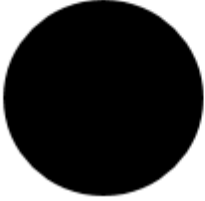 |  |  |

Supplement: Supplementary file 2 [file jmir_v17i2e37_app2.pdf]
